# Supplementary material for: Caffeine Intake throughout Pregnancy, and Factors Associated with Non-Compliance with Recommendations: A Cohort Study
Source: Nutrients. 2022 Dec 18;14(24):5384. doi: 10.3390/nu14245384 (PMC9785327; doi:10.3390/nu14245384)
Supplement: Supplementary file 1 [file nutrients-14-05384-s001.zip › nutrients-2014338-supplementary.pdf]

Table S1. Transformation of beverage and food in mg of caffeine.

Beverage/food	Volume	Mg caffeine
**Coffee**[a]	1 cup 150 ml	100mg
**Decaffeinated coffeea**[a]	1 cup 150 ml	2mg
**Tea**[a]	1 cup 150ml	39mg
**Cola**[a]	1 bottle 200ml	20mg
**Energetic drinks**[b]	1 bottle 200ml	64mg
**Chocolate without milk**[c]	1 square 28g	23mg
**Chocolate with milk**[c]	1 square 28g	6mg

Adapted from: [a]Li et al., 2015; [b]http://energydrink-es.redbull.com/cafeina-en-red-bull; [c]Cnattingius *et al*., 2000
